# *In situ* preparation of a Bi_2_O_2_CO_3_/BiOI with 2D/2D p-n heterojunction photocatalyst for water purification under visible light

**DOI:** 10.3389/fchem.2022.1102528

**Published:** 2023-01-09

**Authors:** Xiaoge Wu, Nan Qin, Lei Yan, Renlong Ji, Di Wu, Zhenhua Hou, Weihua Peng, Jianhua Hou

**Affiliations:** ^1^ College of Environmental Science and Engineering, Yangzhou University, Yangzhou, Jiangsu, , China; ^2^ College of Materials Science and Engineering, Yantai Nanshan University, Longkou, Shandong, China; ^3^ Jiangxi Xinda Hangke New Materials Technology Co., Ltd., Nanchang, China; ^4^ Key Laboratory of Mine Water Resource Utilization of Anhui Higher Education Institutes, Suzhou University, Suzhou, China

**Keywords:** Bi_2_O_2_CO_3_, BiOI, p-n heterojunctions, photocatalysis, water purification

## Abstract

**Introduction:** Semiconductors have similar crystal structures and matched energy levels could form a coupled heterojunction at an interface between them which may allow response to visible light, achieving efficient decomposition of organic compounds.

**Methods:** The Bi_2_O_2_CO_3_/BiOI (BOC/BOI) with 2D/2D p-n heterojunction was prepared by one-pot room-temperature strategy. The prepared materials were tested by various technologies, and the three-dimensional structure, light absorption properties, electrochemical properties and other information were obtained. Photocatalytic tests have also been carried out.

**Results and discussion:** BOC/BOI heterojunction with oxygen vacancies showed much higher photocatalytic activity than pure BOC and BOI. For example, the preferred BOC/BOI-0.5 heterojunction of the degradation rate for Rhodamine B (RhB) is 97.6 % within 2 h, which is 15.8 and 2.2 times faster than that of BiOI and BOC. In addition, the removal rates of tetracycline, ciprofloxacin and bisphenol A by BOC/ BOI-0.5 were 92.4, 80.3 and 68.6%, respectively. The 2D/2D structures of BOC/BOI-0.5 with rich in oxygen vacancies combined p-n junction can effectively inhibit the photoinduced electron-hole pair recombination and increase the production of active free radicals. The O_2_- and h+ are the main reactants, giving the composite catalyst potential for degrading a variety of pollutants.

## 1 Introduction

Rapid industrialization and increasing environmental problems have made organic pollutants a prominent and difficult problem worldwide (Feng et al., 2018; Liu et al., 2018). Photocatalysts have much to offer in dealing with such energy and environmental problems, but the photocatalytic activity of many photocatalysts is by their response range and quantum efficiency (Sakthivel et al., 2020; Xu et al., 2020). It has been reported (Hamza et al., 2020) that the heterostructured photocatalysts such as Bi_2_O_2_CO_3_/Bi_2_WO_6_ (Qiang et al., 2021), g-C_3_N_4_/BiOI (Hou et al., 2021a), BiVO_4_/Ag_3_VO_4_ (Liu et al., 2021b) and BiOCl/Bi2O2CO3 (Hou et al., 2020b) can combine the advantages of two or more semiconductor materials with matching band structures with good light absorption and charge separation.

The 2p valence band of oxygen and bismuth’s 6s valence band make bismuth-containing semiconductors stand out from other heterostructured photocatalysts (Li et al., 2018; Zhang et al., 2020). Bismuth oxyiodide (BiOI) and bismuth carbonate oxide (Bi_2_O_2_CO_3_) with have a Sillén-structure in which the Bismuth, oxygen, iodine or carbonate are orthogonal to each other (Xu et al., 2017), Alternating growth in its crystal structure easily forms layered structures. Different rates of crystal growth facilitate the synthesis of 2D structures which expose specific crystal planes, or 3D structures assembled from 2D structural elements (Chen et al., 2018). A two-dimensional structure, or a three-dimensional structure consisting of two-dimensional sheets exhibits strong photocatalytic activity due to the high surface energy and large specific surface area of such structures (Shi et al., 2018). In addition, the internal electric field formed between the interlaminations can promote the effective separation of photogenerated electrons and holes, which also improves the photocatalytic performance (Di et al., 2017). However, Bi_2_O_2_CO_3_’s wide band gap (2.87–3.58eV) can only be activated under UV irradiation (Zu et al., 2021), and the narrow band gap of BiOI (1.73–1.92eV) also greatly limits its further development in the degradation of organic pollutants (Lu et al., 2021). Considering that BiOI and Bi_2_O_2_CO_3_ have similar crystal structures and matched energy levels, a coupled heterojunction can be formed at an interface between them which may allow response to visible light, improving the effective separation of light-induced electron-hole pairs, improving the adsorption dyestuffs (Qi et al., 2022), and achieving efficient decomposition of organic compounds (Chen et al., 2022).

This study therefore aimed to synthesize Bi_2_O_2_CO_3_/BiOI heterojunction photocatalysts with enhanced photo-responsive activity applying one-pot synthesis at room temperature. The photocatalytic activity of the Bi_2_O_2_CO_3_/BiOI heterojunctions synthesized was tested in Rhodamine B (RhB) degradation. Changing the molar ratio of the reactants altered the photocatalytic performance as expected. And the Bi_2_O_2_CO_3_/BiOI heterojunction catalyst displayed very high photocatalytic activity into RhB degradation under visible light.

## 2 Experiments

### 2.1 Materials preparation

The chemicals used in the catalysts’ preparation—potassium iodide (KI), bismuth nitrate (Bi(NO_3_)_3_⋅5H_2_O), urea (CH_4_N_2_O), nitric acid (HNO_3_) and the scavenging agents ethylenediaminetetraacetic acid disodium salt (EDTA-2Na), isopropyl alcohol and benzoic acid—as well as the Rhodamine B were purchased from Sinopharm Chemical Reagent Co., Ltd. (China). All of the chemicals were of analytical grade.

Bi_2_O_2_CO_3_ was obtained by mixing 970.2 mg of Bi(NO_3_)_3_·5H_2_O with 12 ml of nitric acid, and then adding 200 ml of pure water. The mixture was stirred at 25°C for 0.5 h, and the pH was adjusted to 10 using ammonia solution. 106 mg of Na_2_CO_3_ was then added and stirred for 12 h. The supernatant was removed, centrifuged and washed three times, and the BOC mixture was dried at 60°C.

A similar procedure was involved in the preparation of the BiOI. 970.2 mg of Bi(NO_3_)_3_·5H_2_O was mixed with 12 ml of nitric acid, and then 200 ml of pure water was added. The mixture was stirred at 25°C for 0.5 h, the pH was adjusted to 10 using ammonia solution. 622 mg of KI was then added and stirred at 25°C for 12 h. The supernatant was removed, centrifuged and washed three times, and the resulting BOI mixture was dried at 60°C.

The binary catalyst was synthesized by dissolving 970.2 mg of Bi(NO_3_)_3_·5H_2_O in 12 ml of nitric acid, adding 200 ml of pure water, and stirring at 25°C for 0.5 h. After adjusting the pH to 10 with ammonia, 106 mg of Na_2_CO_3_ was added and the suspension was stirred for 0.5 h. Either 83 mg, 166 mg, 322 mg, 622 mg or 1,244 mg of KI was added, and then sufficient Bi(NO_3_)_3_·5H_2_O was added to make the molar ratio with KI 4:1, 2:1, 1:1, 1:2 or 1:4. The mixture was stirred at 25°C for 12 h, and the supernatant was removed, centrifuged, washed three times, and dried at 60°C. The Bi_2_O_2_CO_3_/BiOI heterojunction catalysts obtained were designated BOC/BOI-4, BOC/BOI-2, BOC/BOI-1, BOC/BOI-0.5 BOC/BOI-0.25 to reflect the molar ratios.

### 2.2 Characterization

X-ray diffraction (XRD) analysis was recorded using a Bruker D8 Advance X-ray diffractometer with Cu K_α_ radiation (*λ* = 1.5406Å) as the X-ray source. Fourier transform infrared spectroscopy (FT-IR) was performed using a TENSOR27 spectrometer. The surface elements were detected using an ESCALAB 250Xi X-ray photoelectron spectroscope (Thermo Fisher Scientific). An A300-10/12 Bruker EPR spectrometer was used to measure the electron paramagnetic resonance (EPR) of the free radicals detected. The morphology and microstructure of the catalyst were examined using a Hitachi, S-4800 scanning electron microscope and high-resolution transmission electron microscopy (HRTEM) was performed with a Tecnai G2 F30 S-Twin instrument. The catalysts’ optical properties were characterized by UV-visual diffuse reflectance spectroscopy relying on a Hitachi U-4100 instrument. A Micromeritics ASAP 2460 instrument was used to record the Brunauer Immett Teller (BET) specific surface areas. A Renishaw inVia confocal Raman microscope was used to study the catalysts’ photoluminescence (PL). Electrochemical impedance spectroscopy (EIS) was performed and Mott Schottky diagrams were prepared in 0.2 M Na_2_SO_4_ electrolyte using a CHI760E electrochemical workstation. The ITO glass electrode was loaded with a sample containing 10 mg of a catalyst, and Ag/AgCl and Pt wires were used to form a three-electrode system. The EIS was over a frequency range of 0.01–10^5^ Hz at 5 mV AC. The frequency of the Mott Schottky test was fixed at 10 Hz. Absorbance readings at 554 nm using a Shimadzu UV-2450 ultraviolet-visible spectrophotometer measured the concentration of RhB.

### 2.3 Photocatalysis tests

For RhB removal, 20 mg of catalyst was mixed with 50 ml of a 20 mg L^−1^ solution of RhB, and the suspension was stirred continuously in the dark for 1 h. The mixture was then illuminated with a 300 W xenon lamp (*λ* > 420 nm), and the 3 ml of the reaction mixture was sampled every 20 min to determine the RhB concentration using the UV-visible spectrophotometer.

In other experiments, 30 mg of the catalyst was mixed with 50 ml of a 10 mg L^−1^ solution of bisphenol A (BPA) and same procedure was followed. Ciprofloxacin (CIP) and tetracycline (TC) degradation were also tested in the same way (Li et al., 2022a).

## 3 Results and discussion

### 3.1 Characterization

**FIGURE 1:**
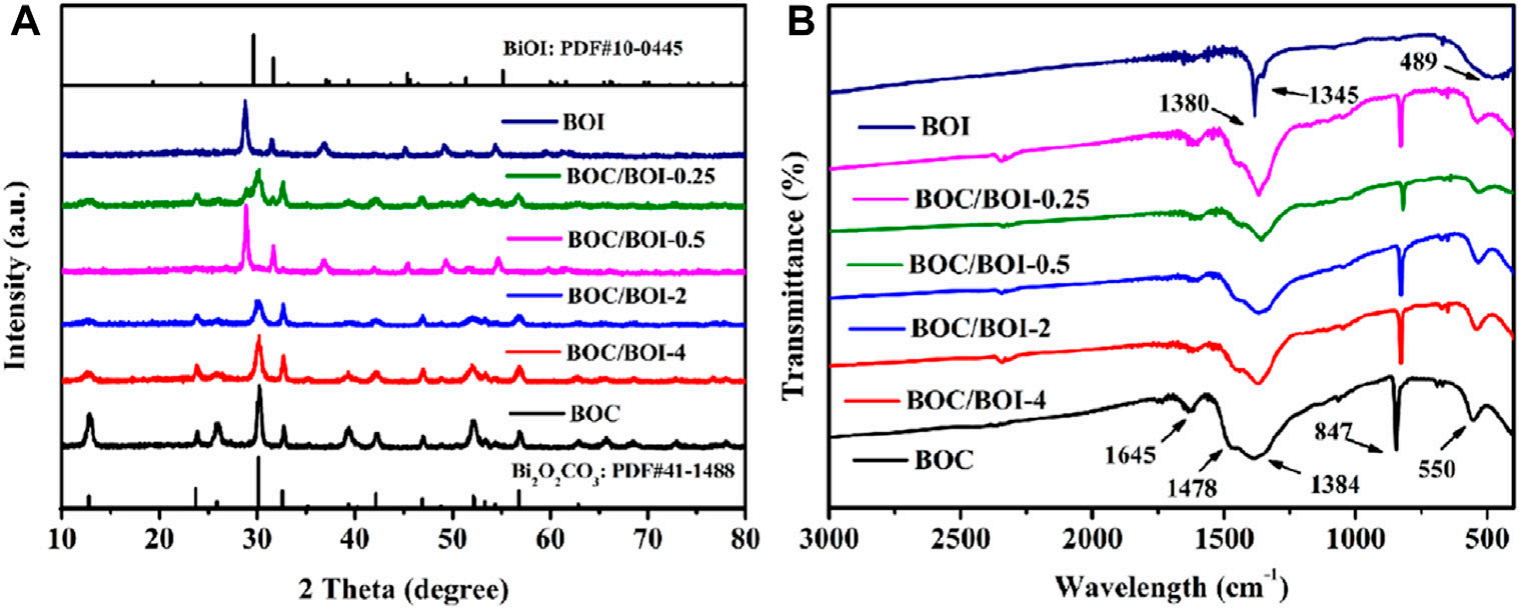
**(A)** XRD patterns, **(B)** FT-IR spectra of all samples.

The prepared samples were characterized using XRD with the results exhibited in Figure 1A. The pure BOC and pure BiOI peaks match the standard cards (JCPDS No. 41-1488 [Qiang et al., 2021) and JCPDS445 (Wang et al., 2017)]. Moreover, the BOC/BOI composite exhibits the coexistence of BOC and BOI phases. The diffraction peak intensity of BOI increases continuously with the KI concentration, while that of Bi_2_O_2_CO_3_ decreases in tandem, indicating that BOC gradually transforms into BOI. The diffraction peak of BOC is barely detectable in the BOC/BOI-0.25 material, indicating that most of the BOC had become BOI.

Infrared spectroscopy was used to explore the chemical composition and chemical bonding of the composites (Li et al., 2022b). Figure 1B presents the FT-IR results. The strong absorption is mainly concentrated in the 400–1750 cm^−1^ region. The bands around 550 and 846 cm^−1^ arise from stretching vibration of Bi-O bonds in BOC, and those around 1,384 and 1478 cm^−1^ are attributable to the stretching vibration of C-O and C=O. The peak at 1,645 cm^−1^ relates to the out-of-plane bending vibration of CO_3_^2-^ (Zu et al., 2021), The peaks of BOI at 1,380 and 1345 cm^−1^ are attributable to the -OH bending vibration of adsorbed H_2_O molecules on the BOI. There is only one peak at about 489 cm^−1^ due to the stretching vibration of Bi-O bonds in BOI (Hou et al., 2021a).

BOC characteristic bands persist in the BOC/BOI composite. As the composite’s BOI content increases, Bi-O bond traces at 550 cm^−1^ gradually move to lower frequencies (compare BOC/BOI-4 to BOC/BOI-0.25). The peak intensities of C-O and C=O at 1384 and 1,478 cm^−1^ also gradually decrease, which proves that BOC and BOI coexist in the composite. No absorption peaks attributable to impurities or solvent residue were detected.

The XPS spectra show the surface chemical states of the photocatalytic materials. The full spectrum survey revealed only Bi, O, C, and N in the BOC. The small amount of N may indicate the presence of BiONO_3_. The excess C content is due to the adsorption of carbon from the air. The curves for BOC/BOI-0.5 in Supplementary Figure S1 indicate the presence of Bi, O, C, and I simultaneously, indicating that BOC and BOI effectively coexist.

**FIGURE 2:**
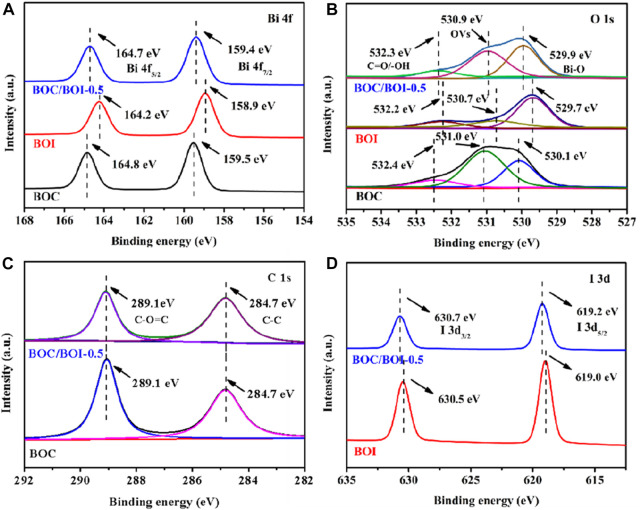
XPS spectra of the **(A)** Bi 4f, **(B)**O 1s, **(C)** C 1s and **(D)** I 3d orbitals of BOC, BOI and BOC/BOI-0.5.

In Figure 2A BOC (159.5 and 164.8 eV) and BOI (158.9 and 164.2 eV) have doublet peaks (attributable to Bi 4f_7/2_ and Bi 4f_5/2_). For BOC/BOI-0.5, the peaks attributable to Bi 4f can be decomposed into 159.4 and 164.7 eV, corresponding to the characteristics of Bi^3+^ (Tang et al., 2020). In Figure 2B, the X-ray photoelectron spectrum, of oxygen’s 1s orbital in BOC/BOI-0.5 can be ascribed to the peak at 529.9 eV due to the Bi-O bond in [Bi_2_O_2_]^2+^ (Ding et al., 2019). The other two characteristic peaks, the possible corresponding oxygen vacancy at 530.9 eV, and the binding energy peak at 532.3 eV, are due to the C=O bond of BOC (532.3 eV) and the surface hydroxyl group of BOI (532.2 eV) (Zhao et al., 2019a). The binding energies of BOC/BoI-0.5 in terms of both the Bi 4f and O 1s orbitals are between those of BOC and BOI. This indicates that Bi_2_O_2_CO_3_/BiOI may have a heterojunction (Li et al., 2022c). For the carbon 1s spectrum (Figure 2C), the characteristic peak of BOC/BOI-0.5 at 284.8 eV corresponds to C-C bonds, and the other characteristic peak (289.1 eV) is from C-O=C (Gao et al., 2021). Compared with BOC, the C 1s peak of BOC/BOI-0.5 is shifted to a lower energy, perhaps due to partial conversion of BOC to BOI. The X-ray spectrum of iodine’s 3d is shown in Figure 2D. The binding energies of BOC/BOI-0.5 are about 619.2 eV (I 3d_5/2_) and 630.7 eV (I 3d_3/2_), indicating that the valence state of iodine is -1 (Cao et al., 2012). And the high energy shift of the I 3d doublet peaks of BOC/BOI-0.5 compared to BiOI (630.5 and 619.0 eV) can also be observed. This is because some of the CO_3_^2-^ ions of the BOC component in BOC/BOI-0.5 have been replaced by I^−^ ions to form BOI, resulting in a corresponding increase in the binding energy of I^−^ in BOC/BOI-0.5 (Zhou et al., 2020). These data verify the existence of both BOC and BOI in BOC/BOI-0.5, and that the binding energy of Bi, O, C and I has shifted compared with BOC and BOI, which confirms the formation of heterojunctions in BOC/BOI-0.5.

**FIGURE 3:**
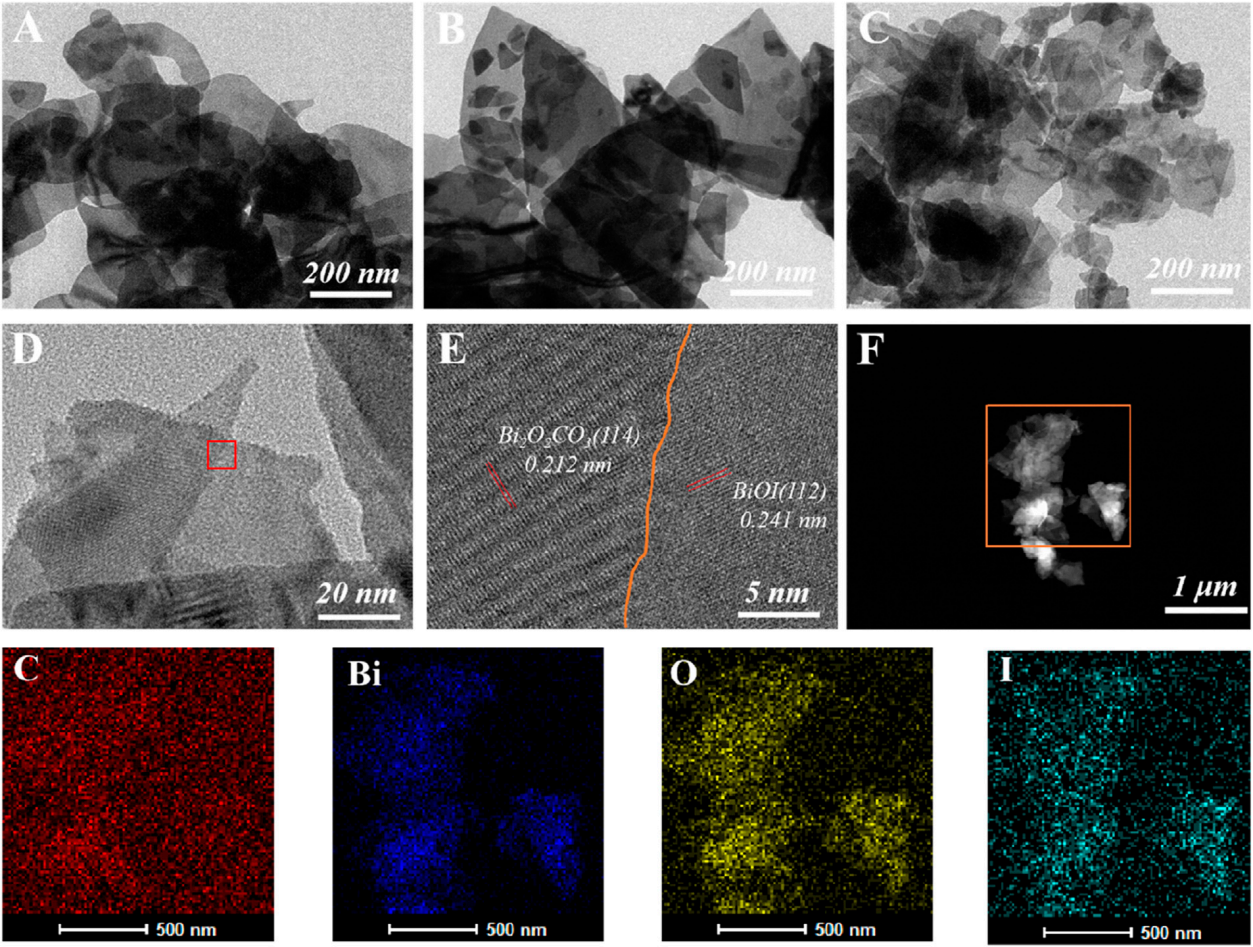
TEM images of **(A)** BOC, **(B)** BOI and **(C)** BOC/BOI-0.5; **(D,E)** high resolution transmission electron micrographs of BOC/BOI-0.5, and **(F)** STEM and element mapping image.

Supplementary Figure S2 presents scanned electron micrographs of BOC, BOI and BOC/BOI-X. All show a 2D-nanosheet structure. Transmission electron micrographs further confirm that BOC/BOI-0.5 is a 2D nanosheet composed of a large number of nanosheets of different sizes (Figures 3C,D). The nanosheets are very thin compared with BOC (Figure 3A) and BOI (Figure 3B), and most of them are translucent under the electron beam, which is consistent with SEM observations. The lattice spacings measured in Figure 3E are 0.212 nm and 0.241 nm, corresponding to the (114) crystal plane of BOC (Feng et al., 2021) and the (112) crystal plane of BOI (Yang et al., 2021), respectively. By analyzing the STEM images (Figure 3F) and the corresponding EDX element mapping of BOC/BOI, the distribution of Bi, O, I and C appears uniform, indicating that BOC and BOI grow effectively coupled, resulting in interfacial interaction and forming 2D/2D heterostructures.

**FIGURE 4:**
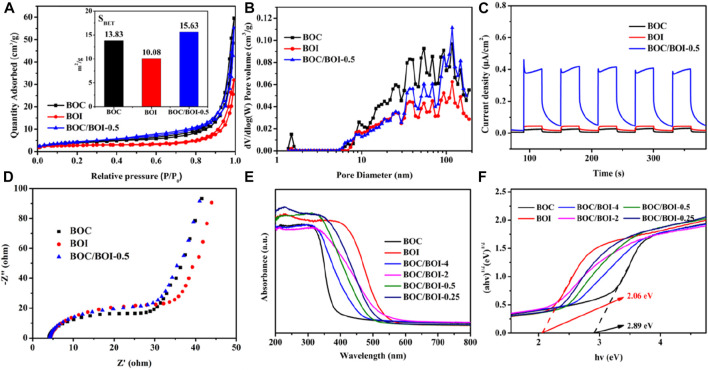
**(A)** Nitrogen adsorption isotherms and BET areas; **(B)** pore size distributions; **(C)** photocurrent; **(D)** Electrochemical impedence spectra of BOC, BOI and BOC/BOI-0.5; **(E,F)** UV-visible diffuse reflectance spectra.

Nitrogen adsorption-desorption experiments were used to study the effect of specific surface area on photocatalytic performance. As shown in Figure 4A, all of the samples tested had type IV absorption curves (Hou et al., 2020a). The specific surface area of the BOC was 13.83 m^2^ g^−1^; that of the BOI was 10.08 m^2^ g^−1^ and for the BOC/BOI-0.5 material it was 15.63 m^2^ g^−1^. The slightly greater specific surface area of the BOC/BOI-0.5 compared to pure BOC and BOI may be due to the coupled growth of BOC and BOI (Liang et al., 2019). In addition, SEM images in Supplementary Figure S2 showed that pure BOC and BOI nanosheets have serious stacking and agglomeration. On the contrary, BOC/BOI-0.5 nanosheets showed oblique angles, resulting in more pore structures. But the data show that the specific surface area of the catalyst did not change significantly, indicating that the specific surface area had no significant effect on the photocatalytic performance. Figure 4B shows the pore size profile. All are in the range from 2 to 120 nm.

The transient photocurrent responses of BOC, BOI and BOC/BOI-0.5 are shown in Figure 4C (Jin et al., 2019). The photocurrent densities of the BOC and BOI electrodes were 0.02 μA cm^−2^ and 0.04 μA cm^−2^, while the photocurrent density of BOC/BOI-0.5 ranged up to 0.40 μA cm^−2^, as much as 20 times greater. The BOC/BOI-0.5 electrode’s high photocurrent density was due to the formation of 2D/2D heterostructures and the high mobility photoinduced e^−^-h^+^ pairs (Tian et al., 2019). Under the same conditions, the enhanced effects of BOC/BOI heterojunctions on interface charge transfer are reflected in the EIS data. As Figure 4D shows, BOC/BOI-0.5 has a smaller Nyquist radius and interface charge transfer resistance in the dark than either BOC or BOI. So coupling BOC with BOI is an effective way to improve the photoinduced e^−^-h^+^ pair separation and interfacial charge transfer (Yu et al., 2018).

The UV-visible diffuse reflectance spectra (Figures 4E, F) further emphasize the fine performance of the BOC/BOI photocatalysts. In Figure 4E, BOC absorbs visible light only weakly, while BOI absorbs almost all of it. The BOC/BOI composites exhibit a blend of the absorption characteristics of BOC and BOI. Since BOC and BOI form a heterojunctions (Li et al., 2023), the strong electric field generated within can accelerate charge transport and facilitate light energy conversion (Jin et al., 2021). The bandgap energy of a semiconductor can be calculated as αhv = A (hv-E_g_) ^n/2^ where *α* is the absorption coefficient, h is Planck’s constant (eV), v is the optical frequency (Hz), E_g_ is the band gap (eV), and A is a fitting a constant. *n* depends on the optical conversion of the semiconductor (*n* = 1 for direct conversion, *n* = 4 for indirect conversion) (Liu et al., 2021a). Bi_2_O_2_CO_3_ and BiOI are indirect bandgap semiconductors (*n* = 4) (Song et al., 2015; Feng et al., 2021). From the plot of (αhv)^1/2^ *versus* (hv) in Figure 4F, the E_g_ of BOC is 2.06 eV and that of BOI is 2.89 eV.

**FIGURE 5:**
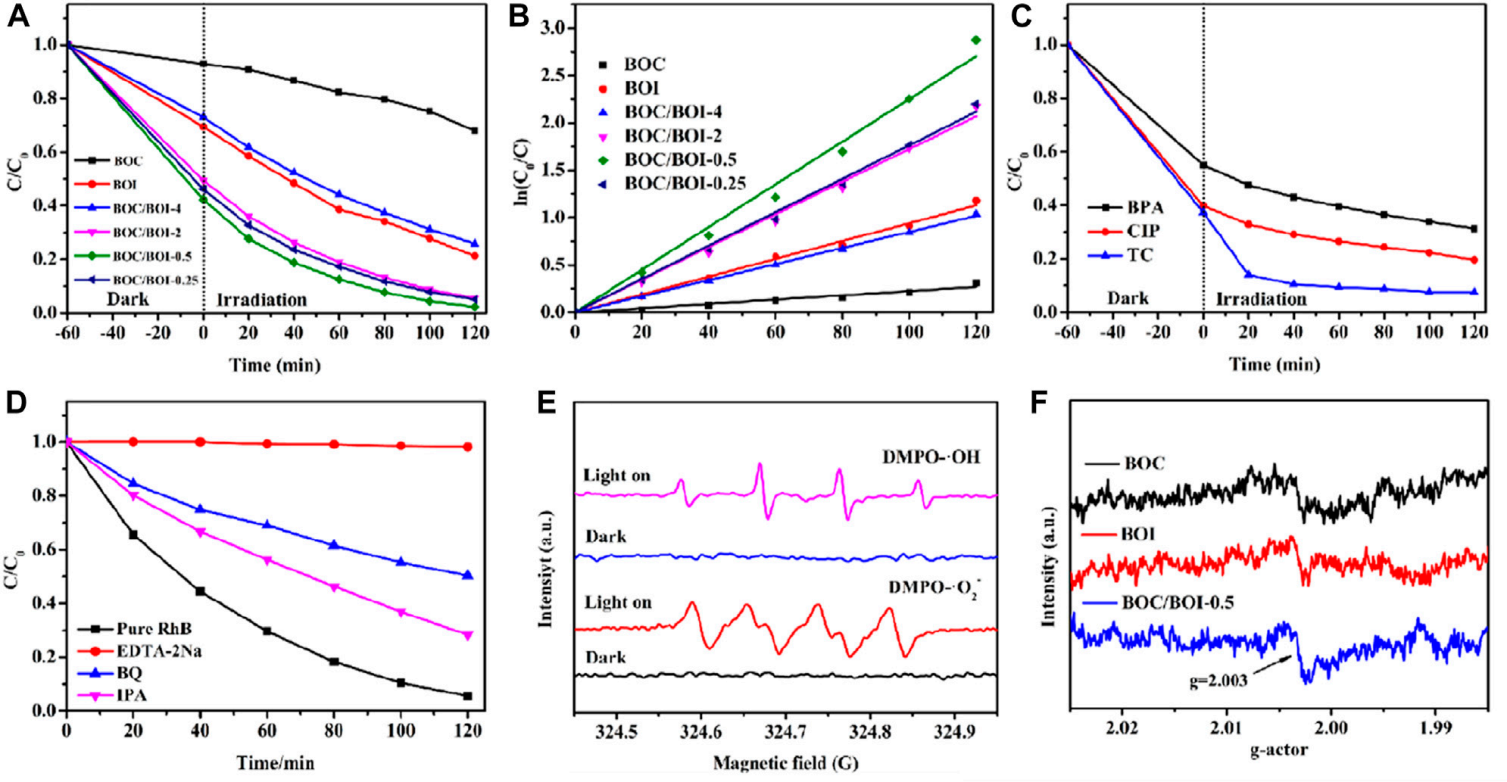
**(A)** Dark adsorption efficiency and photocatalytic degradation of RhB; **(B)** the pseudo-first-order kinetics of each sample; **(C)** dark adsorption and photocatalytic degradation efficiency of other pollutants, **(D)** experimental results of active group capture, **(E)** EPR with ·OH and ·O_2_^−^ as the active group and **(F)** EPR oxygen hole analysis.

The photoelectrochemical properties of the photocatalyst under visible light irradiation were studied in terms of photocatalytic degradation of RhB. Figure 5A shows that the dark adsorption efficiency of BOC/BOI-0.5 was greatly improved compared with BOC or BOI, and the removal of RhB after 2 h was also significantly increased (Wang et al., 2023). The detailed explanation is presented in Section 3.2. It is worth noting, though, that the dark adsorption and photocatalytic effect of BOC/BOI-4 is even worse than that of BOI. However, as the amount of KI increases, the proportion of BOI in the BOC/BOI composite increases, and the dark adsorption and photocatalytic effect gradually increase, reaching an optimum with the best effect at BOC/BOI-0.5. With more KI the photocatalytic performance begins to decrease. In Supplementary Table S1 a linear fit of different BOC/BOI composites for RhB photocatalytic degradation (Figure 5B) suggested that the oxidation reaction kinetics are quasi-first-order (ln (C_0_/C) = kt, *R* ^2^ > 0.99) (Meng et al., 2019). The k value thus estimated for BOC is 0.0025 min^−1^, that for BOI is 0.0096min^−1^ and for BOC/BOI-0.5 the calculation gives 0.024 min^−1^. BOC/BOI-0.5’s photocatalytic degradation rate is almost 10 times that of BOC and 2.5 times that of BOI.

BOC/BOI-0.5’s dark adsorption and photocatalytic degradation were also tested with bisphenol A (BPA), Ciprofloxacin (CIP) and Tetracycline (TC) (Cai et al., 2023). Figure 5C shows that more than 50% of the any of them in 2 h. Tetracycline degradation reached about 90% in 40 min. So BOC/BOI-0.5 is also suitable for treating other organic pollutants. In Figure 5D, EDTA-2Na, benzoic acid and isopropyl alcohol were added to quench h^+^, •O_2_^−^ and •OH, respectively, the main reactive radicals in the photocatalytic process. The quenching greatly inhibited the degradation of RhB was inhibited, so h^+^, •O_2_^−^ and •OH are involved in the photocatalytic process. Among them, h^+^ plays a leading role, probably because photogenerated electrons and holes react with O_2_ and H_2_O to produce •O_2_^−^ and •OH, which then remove pollutants (Liu et al., 2020). EPR tests for active groups (Figure 5E) further confirmed the main active substances in the degradation process. In addition, oxygen holes (Figure 5F) were detected using EPR. Using DMPO spin adducts to capture •O_2_^−^ and •OH radicals, no signal was found under dark conditions, but •OH and •O_2_^−^ radical signal peaks were observed under light irradiation (Liu et al., 2021a). In the EPR oxygen vacancy analysis (Figure 5F), a peak of BOC/BOI-0.5 at g = 2.003 was observed, which proves that there are significant oxygen vacancies in BOC/BOI-0.5 (Zhao et al., 2019b). These results are consistent with those of the quenching experiments, further demonstrating the active groups that play a major role in the photocatalytic process (Geng et al., 2022).

### 3.2 Mechanism

**FIGURE 6:**
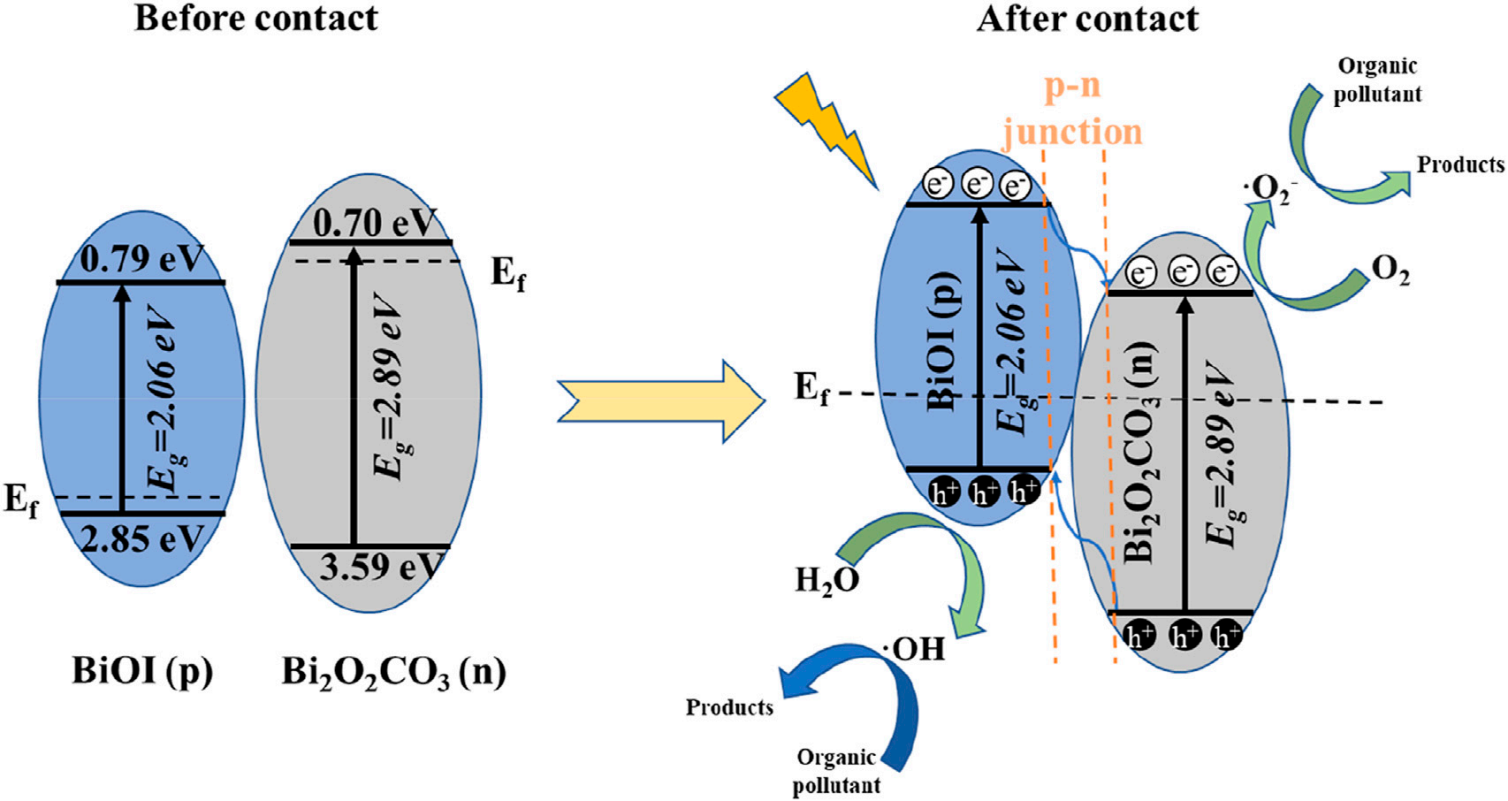
Schematic diagram of the photocatalytic reaction mechanism of Bi_2_O_2_CO_3_/BiOI under visible light irradiation.

Taken together, these results suggest a possible reaction mechanism. The band edge positions of BOC and BOI can be calculated as E_CB_ = X − E_e_ − 0.5E_g_, and E_VB_ = E_CB_ + E_g_ (Song et al., 2015), X here is the electronegativity of the semiconductor, E_e_ is about 4.5 eV, and E_g_ is the bandgap energy (Figure 4F). Using the formulas, the VB of BOC can be calculated as 3.59 eV and its CB as 0.70 eV. For BiOI the values are 2.74 eV and 0.68 eV, and for BOI they are 2.85 eV and 0.79 eV. A schematic diagram of the band structures of BOC and BOI is shown in Figure 6. When irradiated, e^−^ and h^+^ will be generated, and they will interact with oxygen molecules to produce superoxide radical (•O_2_-). Photoinduced h^+^ interacts with water to produce •OH, which is finally decomposed by the interaction between the two radicals and pollutants (Hou et al., 2021b).

Taken together, these findings suggest that the photocatalytic mechanism as below:

i. The nested band structure of Bi_2_O_2_CO_3_ and BiOI seems to be unfavorable for photogenerated carrier separation (Feng et al., 2021). However, BOC and BOI are n-type and p-type semiconductors, respectively, and p-n heterojunction systems can generate a strong electric field at the contact interface due to the large difference in Fermi levels. In an electric field, •O_2_- and •OH will move in opposite directions, which will facilitate separation. Because the Fermi levels of BOC and BOI are very different, after they form a p-n heterojunction the energy band of p-type BOI shifts up along its Fermi level. However, the energy band of n-type BOC moves downward along its Fermi level, which balances the Fermi levels of the BOC and BOI (Lu et al., 2021).
ii. The CB edge potential of BOI is more negative than that of BOC, so the light-induced electrons on the BOI surface easily migrate to the CB of BOC, while the holes are transferred in the opposite direction. The efficiency of separation of the photoinduced carriers is thus greatly improved, which gives the BOC/BOI composite a better ability to degrade pollutants.
iii. Oxygen vacancy (OV) is a kind of metal oxide defects. In this work, it is the separation of oxygen in the lattice caused by metal oxides at high temperature, which leads to oxygen loss and the formation of OVs. The existence of OVs makes the Fermi energy level of oxide move upward, and defect energy level appears in the band gap, thus reducing the band width and improving the light absorption performance. In addition, OVs promote the conversion of excitons into carriers, and accelerate the reduction reaction on the catalyst surface, leading to the separation of carriers. Moreover, the OVs may optimize the adsorption energy of reactants on the catalyst surface, thereby reducing the reaction energy barrier and promoting molecular activation. Thus, OVs plays a synergistic role with nearby active metal sites on the catalyst surface, and photo-generated charge carriers (e^−^-h^+^) can then be effectively separated.
iv. At the same time, e^−^ can participate in the degradation of pollutants by reacting with dissolved oxygen to produce oxidizing •O_2_^−^ free radicals. The h^+^ react with H_2_O (or OH) to produce strongly oxidizing •OH to oxidize organic pollutants. As one of the active substances, h^+^ can also directly oxidize pollutants.
v. RhB can be degraded under the combined action of h^+^, •O_2_^‾^ and •OH.
vi. In the process of RhB degradation, possible sensitization pathways should be considered. RhB may also be excited and inject electrons and residual holes from the excited state RhB* into the heterostructure. The residual holes will then further participate in the degradation reaction (Song et al., 2015). Therefore, the obtained BOC/BOI-0.5 with 2D/2D heterojunction structure is endowed with synergistic effects, which can superior to some other heterojunction materials (as shown in Supplementary Table S2)

## 4 Conclusion

Heterostructured BOC/BOI photocatalytic materials with tunable content have been prepared successfully. The BOC/BOI composite photocatalyst showed greater photocatalytic activity in the degradation of pollutants such as RhB than pure BOC or BOI. The composite’s photodegradation rate of RhB was 9.6 times that of pure BOC. The enhanced photocatalytic activity of BOC/BOI-0.5 largely depends on the p-n heterojunction structure of BOC and BOI. BOC/BOI-0.5 has good interfacial contact and oxygen vacancies. SEM and TEM images confirm that BOC/BOI-0.5 has an ultra-thin nanosheet structure. Photoinduced holes are the main active elements in BOC/BOI-0.5 for the photocatalytic degradation of organic compounds. And BOC/BOI-0.5 also degrades other organic pollutants. It is hoped that in the future this method will be widely applied to the synthesis of other visible light-driven photocatalysts containing bismuth heterostructures with controllable structure.

## Data Availability

The original contributions presented in the study are included in the article/Supplementary Materials, further inquiries can be directed to the corresponding authors.
