# Supplementary material for: In situ preparation of a Bi2O2CO3/BiOI with 2D/2D p-n heterojunction photocatalyst for water purification under visible light
Source: Front Chem. 2023 Jan 9;10:1102528. doi: 10.3389/fchem.2022.1102528 (PMC9870308; doi:10.3389/fchem.2022.1102528)
Supplement: Supplementary file 1 [file DataSheet1.docx]

**Supplementary Information**

**In situ preparation of a Bi_2_O_2_CO_3_/BiOI with 2D/2D p-n heterojunction photocatalyst for water purification under visible light**

Xiaoge Wu ^a *^, Nan Qin^a^, Lei Yan ^a^, Renlong Ji ^b^, Di Wu ^c^, Zhenhua Hou ^c^, Weihua Peng ^d*^, Jianhua Hou ^a, *^

^a^ College of Environmental Science and Engineering, Yangzhou University, Yangzhou, Jiangsu 225127, PR China

^b^ College of Materials Science and Engineering, Yantai Nanshan University, No.12, Daxue Road, Donghai Tourist Resort, Longkou City in Shandong Province 265713, PR China

^c^ Jiangxi Xinda Hangke New Materials Technology Co., Ltd, Nanchang, 330096, China

^d^ Key Laboratory of Mine Water Resource Utilization of Anhui Higher Education Institutes, Suzhou University, Suzhou 234000, PR China

*Corresponding author.

E-mail address: xgwu@yzu.edu.cn (X. Wu); pengweihuapwh@126.com (W. Peng); [jhhou@yzu.edu.cn](mailto:jhhou@yzu.edu.cn) (J. Hou)


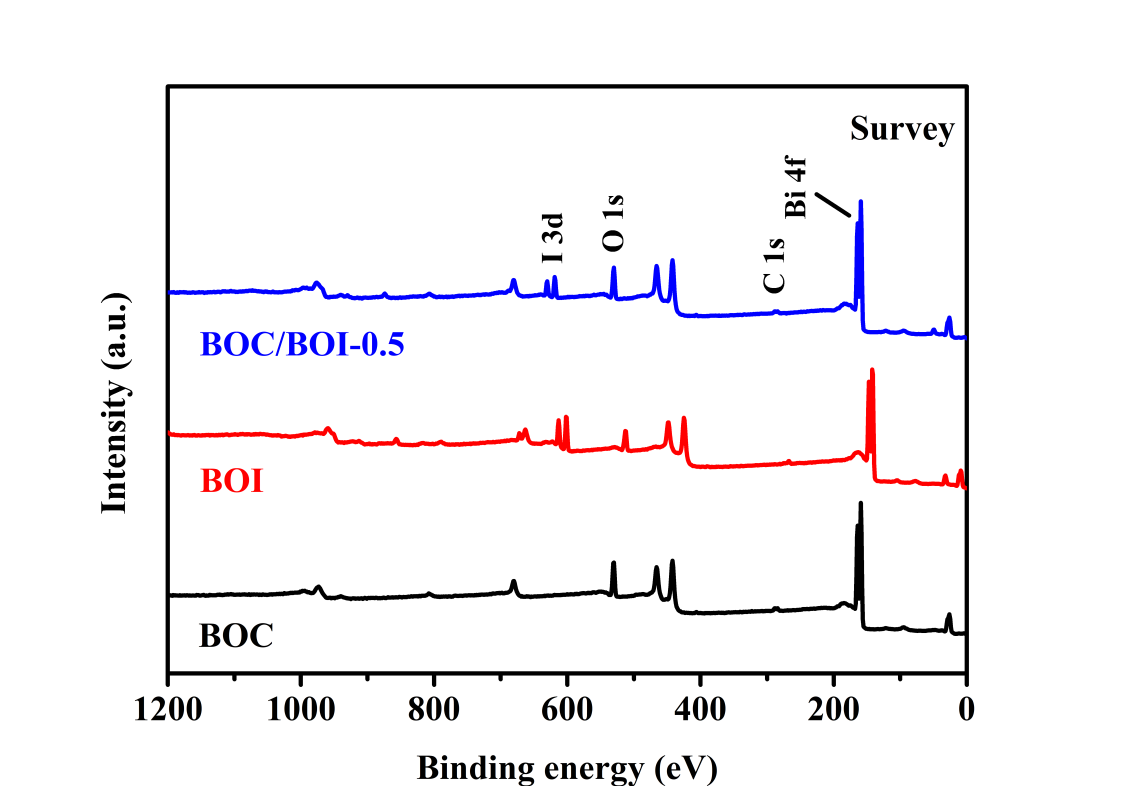


**Figure S1** XPS spectra of full survey.


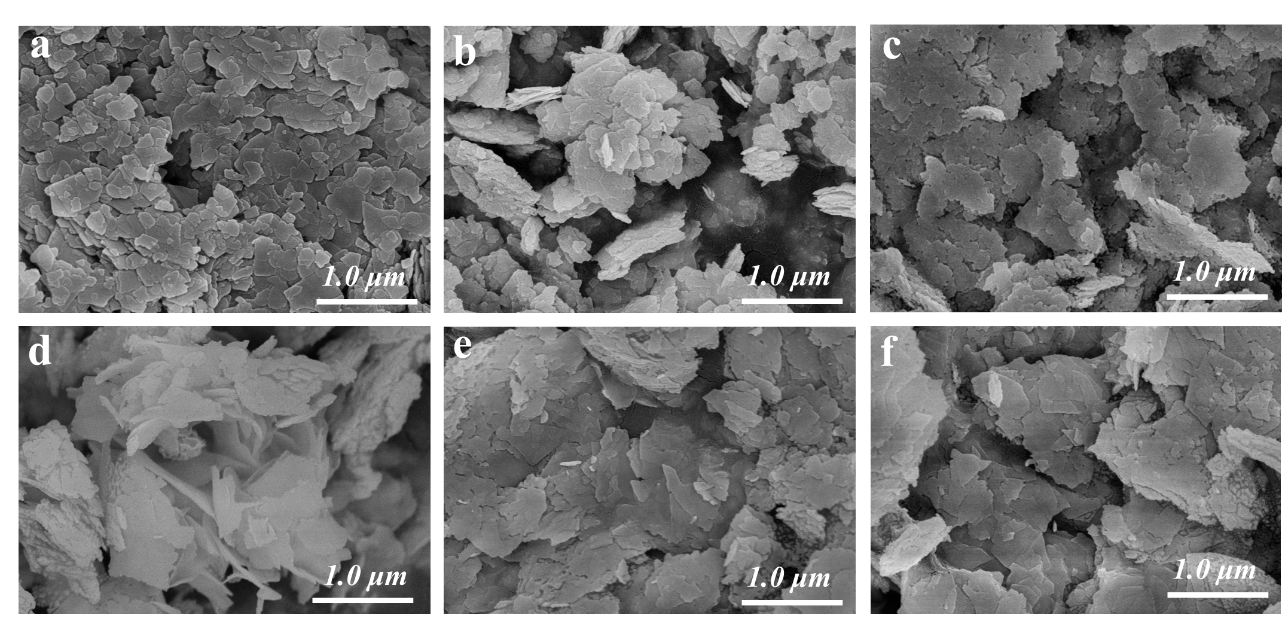


**Figure S2** SEM images of all samples: (a) BOC，(b) BOC/BOI-4，(c) BOC/BOI-2，(d) BOC/BOI-0.5，(e) BOC/BOI-0.25 and (f) BOI

**Table S1** Rate constants and linear fit results for all the samples ^*^ SE: standard errors of k, R^2^: correlation coefficients. RSS: residual sum of squares.

| Materials | Reaction rate constant k (min^-1^) | | SE | R^2^ | RSS |
| --- | --- | --- | --- | --- | --- |
| BOC | 0.0025 | 0.00022 | | 0.9630 | 0.00269 |
| BOI | 0.0096 | 0.00033 | | 0.9943 | 0.00598 |
| BOC/BOI-4 | 0.0086 | 0.00009 | | 0.9994 | 0.00047 |
| BOC/BOI-2 | 0.018 | 0.00058 | | 0.9948 | 0.01879 |
| BOC/BOI-0.5 | 0.024 | 0.00096 | | 0.9917 | 0.05182 |
| BOC/BOI-0.25 | 0.018 | 0.00049 | | 0.9964 | 0.01328 |

**Table S2.** Comparison of Bi_2_O_2_CO_3_/BiOI with other photocatalysts.

| Photocatalyst | C_catalyst_ | C_dye_ | Degradation efficiency | Light source | Ref. |
| --- | --- | --- | --- | --- | --- |
| BiOI/NCC-HCl | 0.6 g·L^-1^ | RhB, 10 mg·L^-1^ | 120 min, ca. 88% | 500 W Xe lamp,  λ > 420 nm | Hou et al., 2019 |
| 1-Ag/ BiOI/pg-C_3_N_4_ | 1.0 g·L^-1^ | MO, 10 mg·L^-1^ | 50 min, ca. 90% | 35 W LED lamp,  λ > 450 nm | Yuan et al., 2020 |
| CeO_2_/g-C_3_N_4_-3 | 1.0 g·L^-1^ | BPA, 10 mg·L^-1^ | 80 min, ca. 94% | 300 W Xe lamp,  λ > 420 nm | Ma et al., 2019 |
| Bi_2_O_2_CO_3_/rGO/PDA | 1.0 g·L^-1^ | MO, 10 mg·L^-1^ | 240 min, ca. 89% | 1000 W Xe lamp,  λ > 420 nm | Su et al., 2020 |
| Bi_4_O_5_Br_2_ HSs | 0.6 g·L^-1^ | TC, 20 mg·L^-1^ | 120 min, ca. 84% | 1000 W Xe lamp,  λ > 420 nm | Bai et al., 2019 |
| Ag_2_MoO_4_/  Bi_4_Ti_3_O_12_ | 1.0 g L^-1^ | TC, 10 mg·L^-1^ | 50 min, ca. 98.7% | 300 W Xe lamp,  λ > 420 nm | Cheng et al., 2021 |
| Bi_4_Ti_3_O_12_/CdS | 1.0 g L^-1^ | MB, 5 mg·L^-1^ | 50 min, ca. 96.3% | 300 W Xe lamp,  λ > 420 nm | Cheng et al., 2022a |
| I_0.4_- Bi_4_Ti_3_O_12_ | 1.0 g L^-1^ | RhB, 5 mg·L^-1^ | 270 min, ca. 99% | 300 W Xe lamp,  λ > 420 nm | Cheng et al., 2022b |
| Bi_5_O_7_I/UiO-66-NH_2_ | 0.75 g·L^-1^ | CIP, 10 mg·L^-1^ | 120 min, ca. 96.1% | 300 W Xe lamp,  λ > 420 nm | Zhao et al., 2021 |
| Cu_3_P-ZnSnO_3_-g-C_3_N_4_ | 0.5 g·L^-1^ | TC, 10 mg L^-1^ | 60 min, ca. 98.45% | 500 W Xe lamp,  λ > 420 nm | Guo et al., 2021 |
| **Bi_2_O_2_CO_3_/BiOI**  **0.4 g·L^-1^** | | **RhB, 20 mg·L^-1^** | **120 min, ca. 97.6%** | **300W Xe lamp,**  **λ > 420 n** | **This work** |
|  |  | **BPA, 10 mg·L^-1^** | **120 min, ca. 68.6%** |  |  |
|  |  | **CIP, 10 mg·L^-1^** | **120 min, ca. 80.3%** |  |  |
|  |  | **TC, 10 mg·L^-1^** | **120 min, ca. 92.4%** |  |  |

(C_catalyst:_ concentration of the catalyst; C_dye:_ concentration of the dye; RhB: rhodamine B, TCH: tetracycline hydrochloride, MO: methyl orange, TC: tetracycline, BPA: bisphenol A, CIP: ciprofloxacin, MB: methylene blue)

**REFERENCES**

Bai, Y., Yang, P., Wang, L., Yang, B., Xie, H.Q., Zhou, Y., Ye, L.Q., 2019. Chem. Eng. J. 360, 473-482.

Cheng, T.T., Gao, H.J., Liu, G.R., Pu, Z.S., Wang, S., Yi, Z., Wu, X.W., Yang, H., 2022a. Colloids Surf. A Physicochem. Eng. Asp. 633.

Cheng, T.T., Gao, H.J., Sun, X.F., Xian, T., Wang, S., Yi, Z., Liu, G.R., Wang, X.X., Yang, H., 2021. Adv Powder Technol. 32(3), 951-962.

Cheng, T. T., Ma, Q., Gao, H. J., Meng, S., Lu, Z., Wang, S., Yi, Z., Liu, G. R., Wang, X. X., Yang, H., 2022b. Mater. Today Chem. 23.

Guo, F., Huang, X.L., Chen, Z.H., Cao, L.W., Cheng, X.F., Chen, L.Z., Shi, W.L., 2021. Sep. Purif. Technol. 265.

Hou, J.H., Jiang, T., Wei, R., Idrees, F., Bahnemann, D., 2019. Front. Chem. 7, 378-387.

Ma, R., Zhang, S., Li, L., Gu, P.C., Wen, T., Khan, A., Li, S.J., Li, B.F., Wang, S.H., Wang, X.K., 2019. ACS Sustainable Chem. Eng. 7, 9699-9708.

Su, M.H., Xu, R.B., Chen, Z.B., Tang, J.F., Ji, C., Yang, M.L., Liu, Y.H., Zhang, H.G., Chen, D.Y., 2020. Ecotox. Environ. Safe.204, 111112.

Yuan, D., Huang, L.Y., Li, Y.P., Wang, H., Xu, X.Q., Wang, C.B., Yang, L., 2020. Dyes Pigments. 177, 108253.

Zhao, C., Li, Y., Chu, H.Y., Pan, X., Ling, L., Wang, P., Fu, H.F., Wang, C.C., Wang, Z.H., 2021. J. Hazard. Mater. 419, 126466.
